# Utilising electrodermal activity sensor signals to quantify nociceptive response during movement activities

**DOI:** 10.1186/s13104-024-06689-9

**Published:** 2024-01-24

**Authors:** Rebecca I. Hamilton, Ashly Alava Garcia, Jake Bowd, David Hamilton, Deborah Mason, Mark Elliott, Cathy Holt

**Affiliations:** 1 Cardiff University, Cardiff, UK; 2 University of Kent, Canterbury, UK; 3 Glasgow Caledonian University, Glasgow, UK; 4 University of Warwick, Coventry, UK

**Keywords:** Electrodermal activity, Sensor data, Nociception, Skin conductance

## Abstract

**Objective:**

With an increasingly ageing population and osteoarthritis prevalence, the quantification of nociceptive signals responsible for painful movements and individual responses could lead to better treatment and monitoring solutions. Changes in electrodermal activity (EDA) can be detected via changes in skin conductance (SC) and measured using finger electrodes on a wearable sensor, providing objective information for increased physiological stress response.

**Results:**

To provide EDA response preliminary data, this was recorded with healthy volunteers on an array of activities while receiving a noxious stimulus. This provides a defined scenario that can be utilised as protocol feasibility testing. Raw signal extraction, processing and statistical analysis was performed using mean SC values on all participant data. The application of the stimuli resulted in a significant average increase (p < 0.05) in mean SC in four out of five activities with significant gender differences (p < 0.05) in SC and self-reported pain scores and large effect sizes. Though EDA parameters are a promising tool for nociceptive response indicators, limitations including motion artifact sensitivities and lack of previous movement-based EDA published data result in restricted analysis understanding. Refined processing pipelines with signal decomposition tools could be utilised in a protocol that quantifies nociceptive response clinically meaningfully.

**Supplementary Information:**

The online version contains supplementary material available at 10.1186/s13104-024-06689-9.

## Introduction

Mechanical loading of osteoarthritic joints results in pain-related functional impairment, causing alterations in joint mechanics, tissue structure and physiological nociceptor interactions [1]. Nociceptive signals are concluded to be the major cause of pain from early to late-stage osteoarthritis (OA) [2]. At present, there are limited options for objective markers of pain experienced by the patient. This consequently affects diagnosis and treatment decisions. Better understanding of pain utilising nociceptive stimuli and response monitoring could lead to better treatment and monitoring solutions [3].

An EPSRC OATech NetworkPlus [EP/N027264/1] funded Sandpit Proof-of-Concept study aimed to develop this notion using currently available technologies resulting in exploratory sensor data results for nociceptive measures [4]. Findings demonstrated a significant noticeable response to a defined thermal stimulus during a stationary standing test. Additional work from our group has investigated the use of electrodermal activity (EDA) in understanding nociceptive pain. The work was the foundations to developing a protocol pipeline to quantify nociceptive response during movement activities.

Of recent years, researchers have begun using EDA for pathophysiological applications like the assessment of fatigue and pain [5]. The paper by Posada-Quintero et al. [5] validated the effectiveness of thermal grills to elicit different levels of pain by using both a subject self-reported VAS and an objective metric of sympathetic neural activities from recordings of EDA. This study evoked high-intensity pain in human volunteers by using safe and non-injurious stimuli via a thermal grill. Posada-Quintero et al. validated the intensity of pain by both subjective measures of subject-reported pain scores and an objective metric of sympathetic neural activities from EDA recordings.

In the current study, the authors incorporated changes in EDA from the activation of the sympathetic nervous system by a noxious stimulus, in the form of increasing temperature. Although questioned in previous method research [5], the quantified stimulus application gives objective benefits that can be developed upon. EDA can be observed as a change in skin conductance (SC), measured in micro siemens (µS). This is composed of skin conductance level (SCL)—background activity of the nervous system, and skin conductance response (SCR)—the activity related to a stimulus.

EDA may be affected by various demographic characteristics, such as those seen between genders [6]. It has been previously reported that females have a greater sweat gland density than males but display more delayed and, in total, less sweating. Bari [7] aimed to investigate gender differences in EDA level (tonic) and responses (phasic) components to some external stimulus via a new non-invasive bioimpedance system, recording EDA measures simultaneously at the same skin site [7]. Although Bari [7] found insignificant gender differences, this paper concluded that it is important to take account of gender to acknowledge potential differences when carrying out studies using EDA measurements.

The aim of this work was to investigate the significance of SC variations in healthy volunteers to provide preliminary data for wider project work investigating a range of wearable sensor data available for detection of EDA nociceptive response.

## Method

SC was recorded for 14 volunteers while performing five activities (stationary standing, sit-to-stand, squat, lunge and 2-step walk) in the Musculoskeletal Biomechanics Research Facility, School of Engineering, Cardiff University. Written informed consent to participate in the study was obtained from all participants. Tests were performed three times during a control condition with no stimuli and a test condition with a thermal stimulus applied (rapid thermal change in temperature of 40–0˚ within a 2 s loop, figure and description in Additional file 1). This was applied to the participants’ right knee using a thermal electrode (Thermal Cutaneous Stimulator, QST.Lab, Strasbourg, France) to define and standardise the nociceptive stimulus. A Visual Analogue Scale (VAS) was used to record self-reported pain scores during each activity.

A battery of exploratory tests was performed on the signals produced. The SC signal was captured using a galvanic skin response (GSR) sensor (Shimmer3 GSR + Unit, Shimmer, Dublin, Ireland). Raw data extraction and pre-processing was conducted using MATLAB. Mean and maximum SC values were used to perform statistical analysis on all participant data.

Testing for normality using a Shapiro Wilk test resulted in abnormal distribution of data and one participant’s data resulted in many outliers. This participant’s data was then removed using standardised values producing Z-scores (values above 2 and less than -2 were removed) to then give normally distributed data with the 13 remaining participants. Mean SC values were compared in paired t-tests using IBM SPSS (V29) with Cohen’s D effect sizes. Mean SC results were compared for the five activities across the control group and intervention group. Further comparisons were then made combining activity data for a male versus female comparison.

## Results

**Fig. 1:**
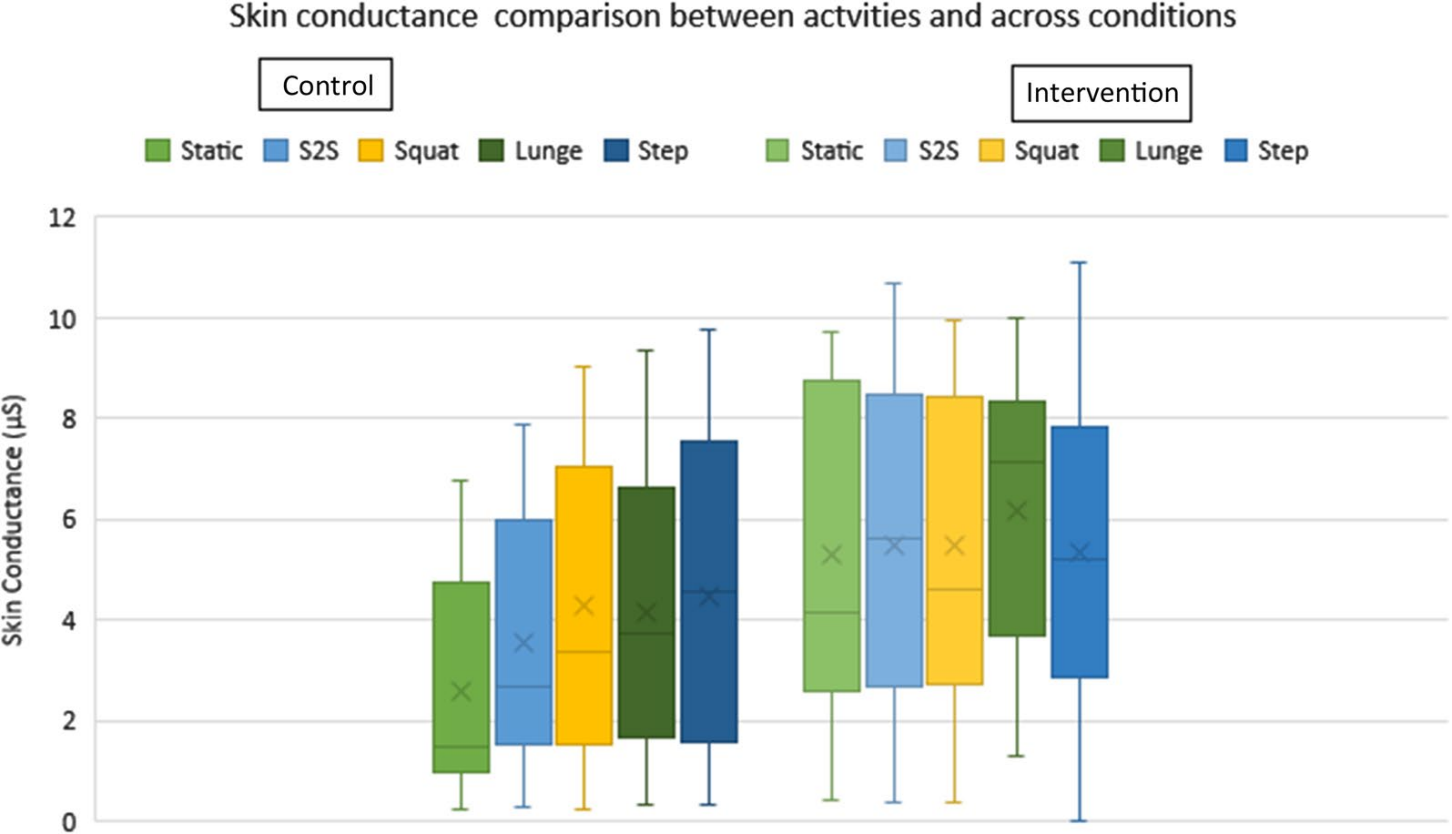
Activity data comparison across both conditions on box plot diagrams displaying data range with median and mean both demonstrated

**Fig. 2:**
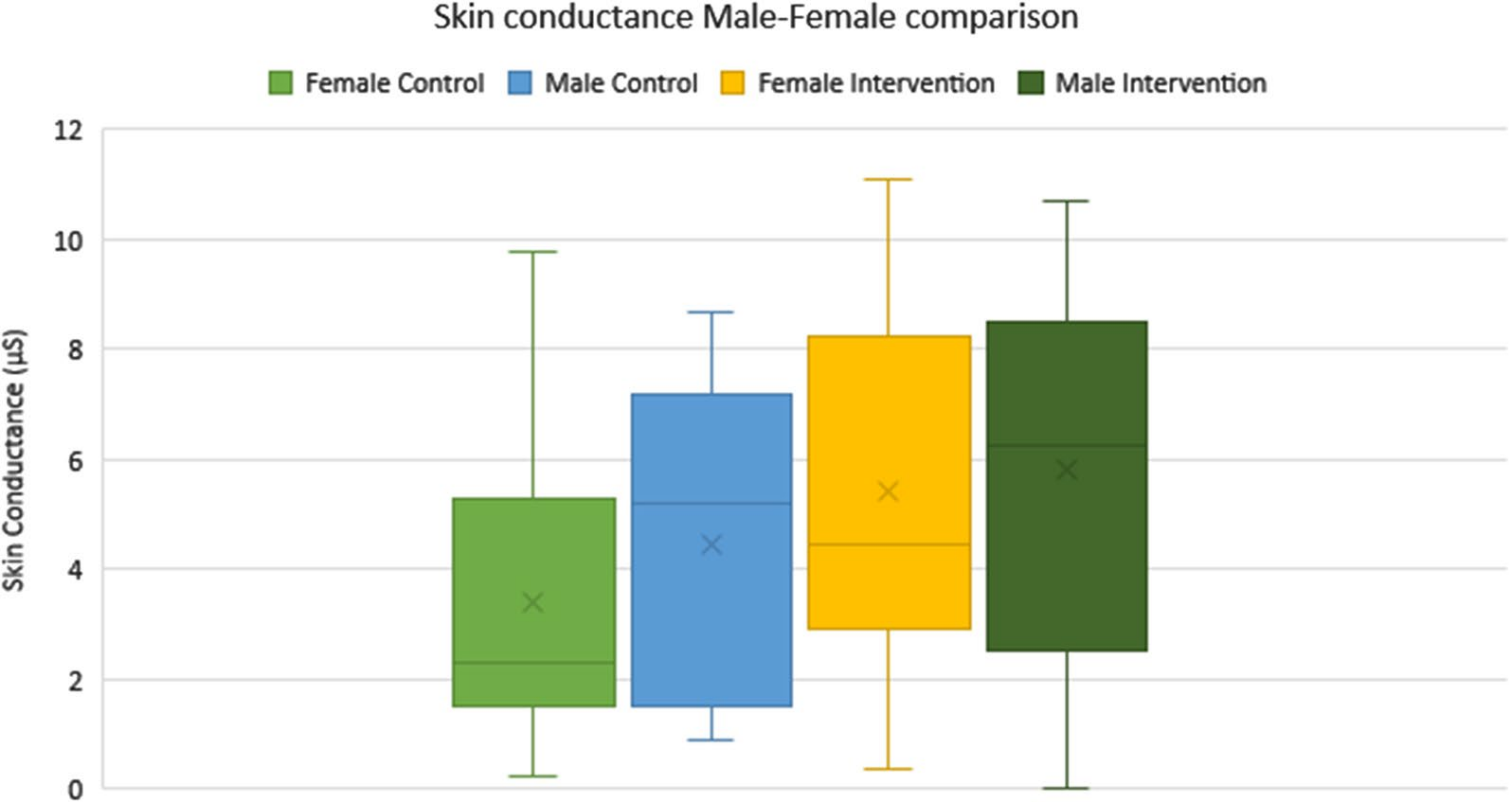
Male and female skin conductance comparison revealing higher values in males compared to females in both control and intervention conditions

The application of the thermal stimuli resulted in a significant increase in mean SC values across initial 13 participants (Females: n = 8, Males: n = 5) during four out of five exercises with high effect sizes (Table 1, Fig. 1), with significant results denoted by *. When activity data was merged into male and female group, a significant difference between genders was found for SC control and VAS intervention comparisons (Table 1, Fig. 2).

**Table 1:** Mean skin conductance and VAS comparisons using paired t-tests and alpha level significance p < 0.05 and statistical significance denoted by*

Comparison	Variable	Activity	P value	Effect size
Control vs Intervention	SC	Static	0.001*	1.08
Control vs Intervention	SC	S2S	0.002*	0.97
Control vs Intervention	SC	Squat	< 0.001*	1.12
Control vs Intervention	SC	Lunge	< 0.001*	0.95
Control vs Intervention	SC	Step	0.282	0.29
Male vs Female	SC	All activities in control condition	0.007*	0.55
Male vs Female	SC	All activities in intervention condition	0.17	0.4
Male vs Female	VAS	All activities in control condition	0.163	0.54
Male vs Female	VAS	All activities in intervention condition	< 0.001*	0.94

## Discussion

Results on all types of analysis revealed increases in SC parameters with the application of a noxious stimulus when compared to a control (Fig. 1, Table 1), providing a compelling case for utilising EDA measures as an indicator for nociceptive response. The results however, should be interpreted with caution based on the study limitations and exploratory nature. Results can be utilised as preliminary data and feasibility testing to build a developed protocol with an EDA data processing pipeline, rather than a direct interpretation of EDA results.

The lack of method refinement for the collection and analysis of EDA data in low intensity exercises is clear from previous literature [5]. Specifically commented, is the frequency range that allows for optimum level of data collection and the use of mean SC versus SCL and SCR data, separating baseline activity and rapid change in response to stimulus. The current study concurs with these findings while utilising mean SC as a feasibility tool to contribute towards a working data collection method. Further decomposition tools will give better granularity in results. With the use of EDA as a response to measures of pain in previous studies show it’s feasible for clinical pain evaluation studies [8], the difficulty of incorporating this into a workable method for patient populations to utilise is still lacking. The current method utilisation of a quantified thermal trigger with off-the-shelf EDA sensors provides a preliminary and feasible method that can be further developed to reduce the limitations on the data.

The significant differences observed between male and female results indicate that there could be some physiological differences in response to pain trigger (males revealing higher SC values) as well as perception of pain (females revealing higher VAS scores). Based on previous gender differences found [7, 9] and whether related to physiological response or perception of pain, it reinforces the need to take gender into account when analysing clinical pain. This could particularly impact pain reporting tools that are the current gold standard method in clinical practice and often do not correlate with clinical disease measures [9].

With motion artifact a known limitation in the data results, the static activity comparison when participants stood still, demonstrates a potential EDA indicator for pain that could be further explored in signal decomposition tools. With many EDA signal outputs referring to SC changes as indicators of stress and pain in stationary situations, there is a clear lack of investigation into GSR sensor data collection during movement and the data processing pipelines in which to do this effectively. Fujita et al. [3] however previously studied changes in SC during different activities, via monitoring with skin impedance electrodes with an OA population and found reductions in response to painful movement, equating to a reduction in skin ability to resist electrical flow and subsequently an increase in SC.

While there is a clear relationship between the applied thermal stimuli and changes in SC, quantifiyng the change and utilising effective signal processing techniques is a considerable challenge due to the difficulty in differentiating between event related activity and baseline activity of the nervous system. More investigation into signal decomposition software and deep learning tools for a more in-depth analysis could help meet this challenge.

## Conclusion

The above findings indicate that nociceptive responses induced by a known pain stimulus can likely be quantified using parameters such as mean SC and number of significant SCR with optimisation techniques. The key findings are: (1) Noticeable significant increase in mean SC during the application of stimuli were observed. (2) Higher values of SC were observed in male participants in comparison to female participants for both conditions. (3) Further analysis and techniques should be explored to optimise and refine data collection and signal processing to select key features for nociceptive EDA response across subject cohort groups.

## Limitations

Interpretation of sensor data is limited by the sensor sensitivity to motion artifact. Incorporating measures that account for this to determine the true EDA values and their level of change due to the noxious trigger is required for these methods to meaningfully develop. There is limited previous published data on GSR sensor outputs during movement activities and therefore no pipeline or protocols for this currently exist for comparison. Thus, all data collected in this field is exploratory. Different processing tools may arise in many ways of interpreting data, decreasing options of standardising data outputs. There are feasibility limitations incorporating this sensor data exploration into a nociceptive response protocol due to the limited technology available and therefore incorporating into clinically meaningful analysis.

### Supplementary Information

**Additional file 1.** Supplementary Material file has been provided which contains an image of the thermode configuration and the MATLAB script used for raw data extraction and pre-processing. 

## Data Availability

Data provided in are summary data and in the Additional file 1. Information on the data underpinning the results presented here, including how to access them, can be found in the Cardiff University data catalogue at 10.17035/d.2024.0305413313.
