# Supplementary material for: Utilising electrodermal activity sensor signals to quantify nociceptive response during movement activities
Source: BMC Res Notes. 2024 Jan 24;17:36. doi: 10.1186/s13104-024-06689-9 (PMC10809522; doi:10.1186/s13104-024-06689-9)
Supplement: Supplementary file 1 — Additional file 1. Supplementary Material file has been provided which contains an image of the thermode configuration and the MATLAB script used for raw data extraction and pre-processing. [file 13104_2024_6689_MOESM1_ESM.docx]

**Supplementary Material**

**Thermode Configuration Figure**


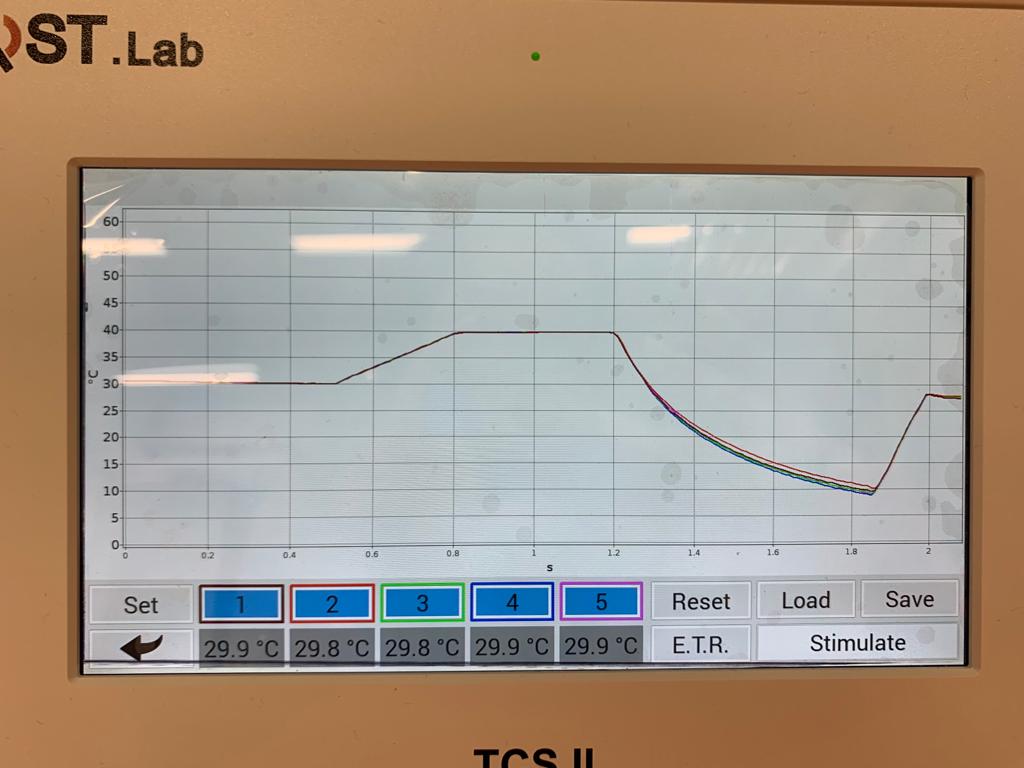


2 second temperature loop started at 30˚C for 0.5seconds, rose to 40˚C over 0.3 seconds and stayed at this temperature for 0.4 seconds before decreasing to 10˚C over 0.6 seconds then raising back up to 30˚C over 0.2 seconds, creating the 40-10˚ temperature loop (see figure in Supplementary Material).

**MATLAB Script to extract summary values from raw EDA data**

%% SEARCH FILE - SCRIPT HAS TO BE IN THE FOLDER AS FILE

clear all; close all;

[file,path] = uigetfile('*.csv','Select One or More Files','MultiSelect', 'on');

partData = xlsread(file);

%% INPUT FREQUENCY

F = 100;

%% CHANGE EACH TIME

trial = 'Outputs_D20_07_2022_test7_Session5_Shimmer_STS_thermode';

% CHANGE EACH TIME

%% time and timestamping

t = 1/F;%time in between recordings

count_rows = length(partData(3:end,1));%count number of rows in file

R = zeros(count_rows,1);%create the matrix for timestamps

R(1,1) = 0;

R(2,1) = R(1,1) + t;

nextrow= 3;

for i = 1:count_rows-2;

R(nextrow,1)= R(nextrow - 1,1) + t;

nextrow = nextrow + 1;

end

t_time = seconds(R(nextrow-1,1));

% CONVERT INTO TIME DURATION

t_time.Format = 'hh:mm:ss';

%% EXTRACT GSR AND TIME COLUMNS

parTime = transpose(R);

parGSR = transpose(partData(3:end,3));

%% INPUT DOWNSAMPLING RATE

dTime = downsample(parTime,6);

dGSR = downsample(parGSR,6);

%% CREATE STRUCT ARRAY

field1 = 'conductance'; value1 = dGSR;

field2 = 'time'; value2 = dTime;

field3 = 'timeoff'; value3 = [];

field4 = 'event'; value4 = []; %to add event markers

data = struct(field1,value1,field2,value2,field3,value3,field4,value4);

%% DETREND DATA

sdata = data.conductance;

detrend_sdata = detrend(data.conductance);

trend = sdata - detrend_sdata;

% PLOTS

figure;

hold on

plot(data.time, data.conductance);

plot(data.time, trend, ':r'); plot(data.time,detrend_sdata,'m'); plot(data.time,zeros(size(dTime)),':k');

title('Raw skin conductance values');

xlabel('time');

ylabel('Raw skin conductance');

legend('raw data','mean','detrended raw data');

saveas(gcf,num2str(trial),'png')

%% STATS CALCULATIONS

avg = mean(parGSR);

SD = std(parGSR);

CV = (SD/avg)*100;

T = table(t_time, avg, SD, CV)

%%% SKIN CONDUCTANCE MAX/MIN/RANGE

[SC_Max_Value, index] = max(data.conductance);

SC_Max_Value_Time_Point = data.time(index);

[SC_Min_Value, index] = min(data.conductance);

SC_Min_Value_Time_Point = data.time(index);

SC_Range = range(data.conductance);

SC_Table = table(SC_Max_Value, SC_Min_Value, SC_Max_Value_Time_Point, SC_Min_Value, SC_Min_Value_Time_Point, SC_Range);

writetable(SC_Table,'Outputs_D20_07_2022_test7_Session5_Shimmer_STS_thermode.xlsx') % USE SAME FILE NAME AS 'trial'
